# Functional toxicology: tools to advance the future of toxicity testing

**DOI:** 10.3389/fgene.2014.00110

**Published:** 2014-05-05

**Authors:** Brandon D. Gaytán, Chris D. Vulpe

**Affiliations:** Department of Nutritional Science and Toxicology, University of California BerkeleyBerkeley, CA, USA

**Keywords:** yeast, toxicology, toxicity testing, functional toxicology, functional genomics, functional profiling

## Abstract

The increased presence of chemical contaminants in the environment is an undeniable concern to human health and ecosystems. Historically, by relying heavily upon costly and laborious animal-based toxicity assays, the field of toxicology has often neglected examinations of the cellular and molecular mechanisms of toxicity for the majority of compounds—information that, if available, would strengthen risk assessment analyses. Functional toxicology, where cells or organisms with gene deletions or depleted proteins are used to assess genetic requirements for chemical tolerance, can advance the field of toxicity testing by contributing data regarding chemical mechanisms of toxicity. Functional toxicology can be accomplished using available genetic tools in yeasts, other fungi and bacteria, and eukaryotes of increased complexity, including zebrafish, fruit flies, rodents, and human cell lines. Underscored is the value of using less complex systems such as yeasts to direct further studies in more complex systems such as human cell lines. Functional techniques can yield (1) novel insights into chemical toxicity; (2) pathways and mechanisms deserving of further study; and (3) candidate human toxicant susceptibility or resistance genes.

## Chemical production and its implications

Current estimates project that global chemical production—currently growing 3% per year—will double every 25 years (Wilson et al., 2006). In the United States alone, excluding fuels, pesticides, pharmaceuticals, or food products, about 42 billion pounds of chemicals are produced or imported daily (U.S. EPA, 2005). Many chemicals are managed through the Toxic Substances Control Act (TSCA), but several independent analyses have concluded that these regulations seriously hinder (1) toxicity testing and hazard assessment; (2) control of chemicals of concern; and (3) investment in safer alternatives, such as those generated by the tenets of green chemistry (Wilson and Schwarzman, 2009). Combined with the widespread use and distribution of industrial chemicals, the data and safety gaps precipitated by TSCA elicit a situation in which chemical exposures to humans and ecosystems are oftentimes of unknown hazard and risk.

## The present state of chemical toxicity testing

The field of toxicology currently employs extensive animal-based assays to evaluate chemical toxicity, a burdensome and prohibitively expensive approach that typically assesses a limited number of endpoints. Considering that tens of thousands of in use chemicals lack adequate toxicity data (Judson et al., 2009), it is unreasonable to rely upon these traditional methods to fill data gaps. The National Research Council (NRC), realizing that more innovative approaches to testing were needed, envisioned that toxicology should commit to mechanistically-based high-throughput cellular *in vitro* assays (NRC, 2007; Andersen and Krewski, 2010). In this way, a more complete comprehension of chemical toxicity can be achieved, while expediting testing, decreasing costs, and reducing animal usage.

Although high-throughput *in vitro* methods certainly signal progress in toxicity testing, they are limited to existing assays with known endpoints, such as analyses of stress response pathways induced by oxidative species, heat shock, DNA damage, hypoxia, and unfolded proteins (Simmons et al., 2009). Another approach utilizes “omics” technologies such as gene expression profiling, proteomics, lipidomics, and metabolomics to conduct targeted and untargeted investigations into chemical mechanisms of toxicity (reviewed by Hamadeh et al., 2002; Gatzidou et al., 2007). However, by associating toxicant exposure with changes in mRNA, protein, lipid, or metabolite levels, these assays are correlative and do not provide direct links between genes and their requirements in the cellular toxicant response.

## The advantages of functional toxicology

**Figure 1:**
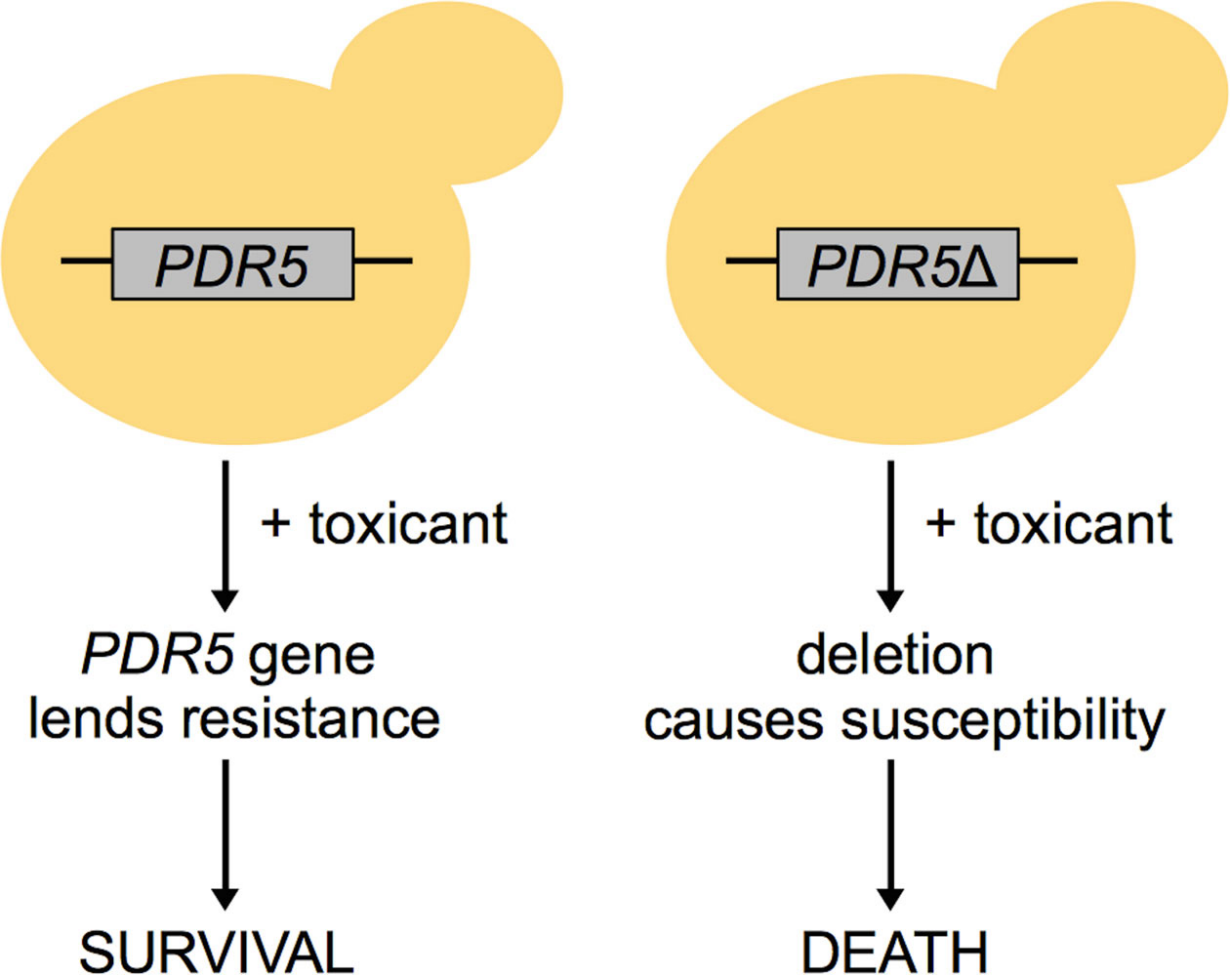
**The concept of functional toxicology in yeast**. In this example, a yeast cell with the *PDR5* gene is able to survive under toxicant selection, whereas a cell deleted for *PDR5* experiences susceptibility to that same toxicant. Therefore, the *PDR5* gene is essential for survival in that toxicant.

Functional toxicology is based in the high-throughput use of cells/organisms harboring gene deletions or depleted proteins to systematically examine genetic requirements for toxicity tolerance. Any assayable phenotype can be measured in response to a toxicant, but viability or fitness are the most conventional endpoints (Figure 1). Functional techniques can provide information distinct from the aforementioned correlative methodologies; for example, Giaever et al. (2002) found that expression of a gene is generally unrelated to its requirement for growth under a selective condition. Functional analyses, which have been conducted in budding and fission yeast (Table 1), bacteria, nematodes, fruit flies, zebrafish, and human cell lines (Table 2), can (1) contribute novel insight into chemical mechanisms of action; (2) define more specific toxicological endpoints; and (3) inform further mechanistic-based assays.

**Table 1:** **Summary of recent functional toxicological screens in yeasts**.

**Chemical class**	**Description**	**Organism**	**References**
Solvents	Butanol	*S. cerevisiae*	González-Ramos et al., 2013
	Dimethylsulfoxide	*S. cerevisiae*	Zhang et al., 2013
	Dimethylsulfoxide	*S. cerevisiae*	Gaytán et al., 2013a
Metals	Aluminum	*S. cerevisiae*	Tun et al., 2013
	Gold nanoparticles	*S. cerevisiae*	Smith et al., 2013
	Cobalt	*S. pombe*	Ryuko et al., 2012
	Cadmium	*S. pombe*	Kennedy et al., 2008
Persistent pollutants	Dieldrin	*S. cerevisiae*	Gaytán et al., 2013b
	Toxaphene	*S. cerevisiae*	Gaytán et al., 2013c
Antimicrobials	2,4-diacetylphloro- glucinol	*S. cerevisiae*	Troppens et al., 2013
	Antimicrobial peptides	*S. cerevisiae*	Lis et al., 2013
	Curcumin	*S. cerevisiae*	Azad et al., 2013
	Chitosan	*S. cerevisiae*	Galván Márquez et al., 2013
	Eugenol	*S. cerevisiae*	Darvishi et al., 2013a
	Thymol	*S. cerevisiae*	Darvishi et al., 2013b
	Polyalkyl guanidiniums	*S. cerevisiae*	Bowie et al., 2013
	TA-289	*S. cerevisiae*	Quek et al., 2013
	Micafungin	*S. pombe*	Zhou et al., 2013
	Various antifungals	*S. pombe*	Fang et al., 2012
Drugs	Chloroquine	*S. cerevisiae*	Islahudin et al., 2013
	Edelfosine	*S. cerevisiae*	Cuesta-Marbán et al., 2013
	Porphyrin TMpyP4	*S. cerevisiae*	Andrew et al., 2013
	FK506	*S. pombe*	Ma et al., 2011
	Caffeine	*S. pombe*	Calvo et al., 2009
Genotoxicants	Methyl methanesulfonate	*S. cerevisiae*	Huang et al., 2013b
	Various	*S. cerevisiae*	Svensson et al., 2013
	Various	*S. cerevisiae*	Torres et al., 2013
	Various	*S. pombe*	Pan et al., 2012
Other	Acetic acid	*S. cerevisiae*	Sousa et al., 2013
	Hydrolysate	*S. cerevisiae*	Skerker et al., 2013
	Neonicotinoids	*S. cerevisiae*	Mattiazzi Ušaj et al., 2013
	Manzamine A	*S. cerevisiae*	Kallifatidis et al., 2013
	NCI diversity/ mechanistic sets	*S. cerevisiae/ S. pombe*	Kapitzky et al., 2010

A literature search identified recently published S. cerevisiae and S. pombe screens.

**Table 2:** **Summary of functional toxicological screens in organisms other than yeast**.

**Chemical/condition**	**Organism**	**Methodology**	**References**
Clotrimazole	*C. albicans*	Deletions	Oh et al., 2010a
Wide variety of growth conditions and diverse chemical compounds	*C. albicans*	Deletions	Oh et al., 2010b
Paraquat	*C. elegans*	RNAi	Kim and Sun, 2007
Cell cycle inhibitors	*D. melanogaster*	RNAi	Eggert et al., 2004
Cell cycle inhibitors	*D. rerio*	Morpholinos	Murphey et al., 2006
Various media and growth inhibitors	*E. coli*	Deletions	Warner et al., 2010
Vemurafenib	*H. sapiens*	CRISPR	Shalem et al., 2014
6-thioguanine	*H. sapiens*	CRISPR	Wang et al., 2014
Etoposide	*H. sapiens*	CRISPR	Wang et al., 2014
Wide range of drugs	*H. sapiens*	RNAi	reviewed by Berns and Bernards (2012)
Ricin	*H. sapiens*	shRNA	Bassik et al., 2013
3-bromopyruvate	*H. sapiens*	Transposon mutagenesis	Birsoy et al., 2013
Tunicamycin	*H. sapiens*	Transposon mutagenesis	Reiling et al., 2011
2-amino-6-mercaptopurine	*M. musculus*	Transposon mutagenesis	Leeb and Wutz, 2011
6-thioguanine	*M. musculus*	Transposon mutagenesis	Pettitt et al., 2013
Olaparib	*M. musculus*	Transposon mutagenesis	Pettitt et al., 2013
Ricin	*M. musculus*	Transposon mutagenesis	Elling et al., 2011
Wide variety of growth conditions and diverse chemical compounds	*S. oneidensis*	Deletions	Deutschbauer et al., 2011
Minimal media	*S. oneidensis*	Deletions	Oh et al., 2010a
Plant hydrolysate	*Z. mobilis*	Deletions	Skerker et al., 2013

A literature search identified published screens.

## Functional toxicology in yeasts

For many reasons, the eukaryotic budding (*Saccharomyces cerevisiae*) and fission (*Schizosaccharomyces pombe*) yeasts are ideal models in which to conduct functional toxicological studies. Numerous metabolic and signaling pathways, along with basic cellular processes, are conserved with more complex organisms such as humans. Human homologs have been identified for a large number of yeast genes, with several hundred of the conserved genes linked to disease in humans (Steinmetz et al., 2002; Wood et al., 2002). A long history of genetic manipulation in yeasts confers the ability to selectively target and examine conserved genes and pathways throughout their genomes, facilitating functional analyses. The ease of culture and availability of software resources, molecular protocols, and genetic and physical interaction data collectively bolster the value of yeasts in toxicology.

**Figure 2:**
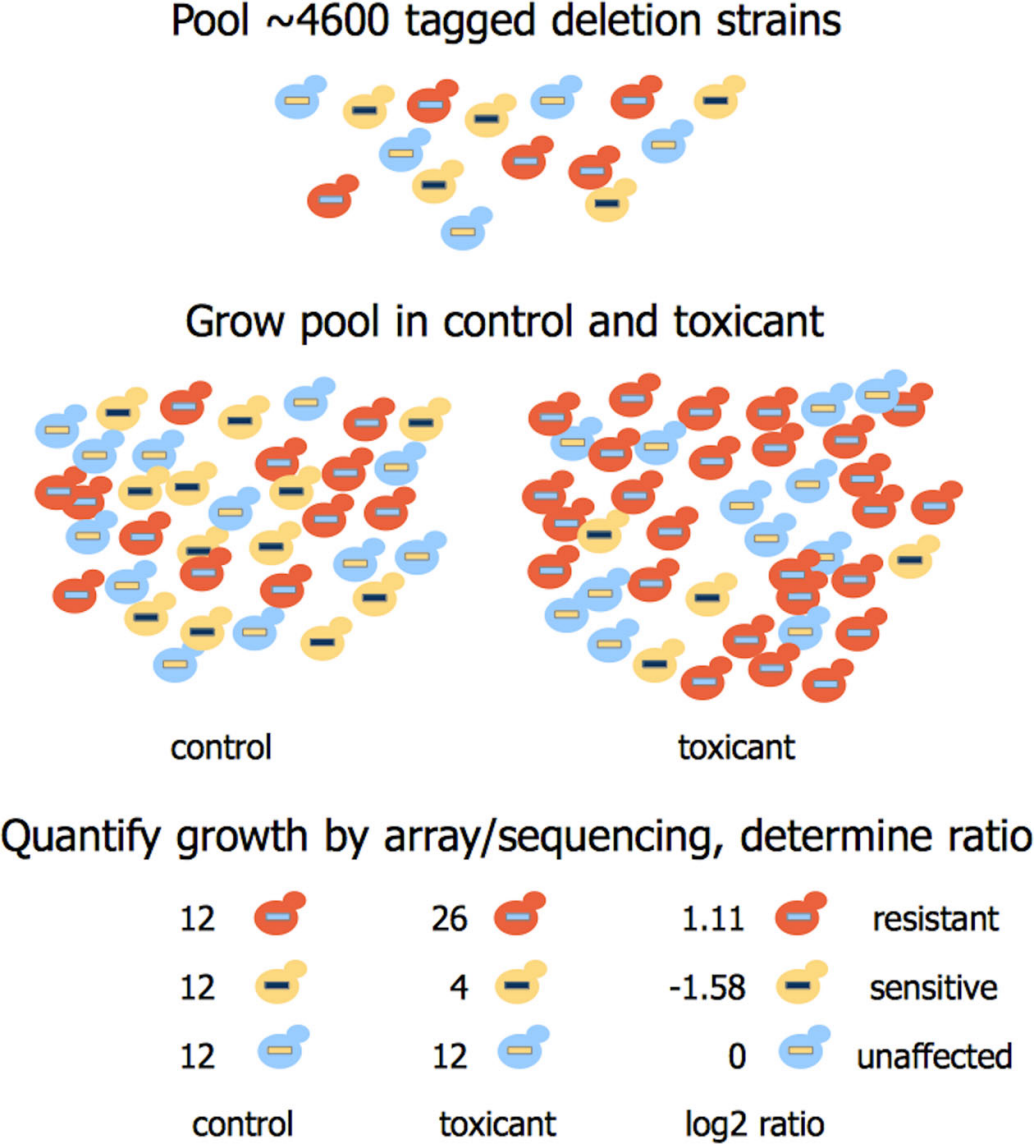
**Overview of functional profiling in yeast**. About 4600 deletion strains uniquely identified by DNA sequences (barcodes) are pooled and exposed to a toxicant at multiple doses and generation times (5 or 15). Barcodes are amplified from purified genomic DNA by PCR and counted by hybridization to a microarray or high-throughput sequencing methods. Subsequent analyses of individual strains can confirm susceptibility or resistance to the toxicant.

Barcoded mutant collections have been generated in budding (Giaever et al., 1999, 2002) and fission yeast (Kennedy et al., 2008; Kim et al., 2010; Chen et al., 2012), allowing assessment of individual strain fitness in pooled cultures under selective conditions (reviewed by North and Vulpe, 2010; dos Santos et al., 2012). This technique, known as functional profiling, functional genomics, chemical genomics, or chemical-genetic profiling, can identify the genetic requirements for tolerance to any substance that causes measurable growth inhibition in yeast. Figure 2 demonstrates the screening process, while Table 1 provides a summary of recent functional studies. Homozygous profiling (HOP) utilizes strains deleted for non-essential genes to establish the genetic requirements for chemical tolerance, while haploinsufficiency profiling (HIP) detects strains sensitized to a chemical targeting the product of their corresponding heterozygous locus (for a review, see Smith et al., 2010a). In brief, DNA sequences (“barcodes”) uniquely identifying each deletion strain enable parallel growth analyses with thousands of pooled mutants exposed to a chemical of interest. A PCR amplification of the barcodes and their subsequent quantification via microarray hybridization or sequencing allows for discovery of strains with altered growth in the particular substance. The decreased abundance by mRNA perturbation (dAMP) collection complements heterozygote profiling by destabilizing a gene's mRNA (and thus depleting the encoded protein) via disruption of 3′-untranslated regions (Yan et al., 2008). An additional tool that may benefit functional toxicology is the barcoded yeast overexpression library (Ho et al., 2009; Douglas et al., 2012). Similar to HOP and HIP, this technique enables highly parallel and systematic investigations of overexpression phenotypes in pooled cultures. Finally, a novel “functional variomics” approach utilizes high-complexity random mutagenesis to identify genes conferring drug resistance due to mutations or overexpression (Huang et al., 2013a). The advent of multiplexed high-throughput barcode sequencing of pooled cultures (Han et al., 2010; Smith et al., 2010b) promises a future of expedited and cost efficient functional genomic analyses.

The functional tools available in yeast provide unmatched resources for inquiries into potential cellular and molecular mechanisms of toxicity. Such analyses have informed functional experimentation in more complex organisms such as zebrafish or human cell lines. For example, Ishizaki et al. (2010) utilized a yeast chemical-genetic screen to reveal that intracellular trafficking defects conferred sensitivity to copper limitation, and further reported that knockdown of zebrafish homologs to these yeast genes sensitized fish to copper-dependent hypopigmentation, a hallmark of copper deficiency in humans. Following identification of the Sas2p histone acetyltransferase as a modulator of arsenite tolerance in yeast, knockdown of its homolog *MYST1* in human bladder epithelial cells was found to similarly induce arsenite sensitivity (Jo et al., 2009a,b). Another group demonstrated that the investigational cancer drug elesclomol affected electron transport mutants in yeast and extended their analysis by determining that elesclomol interacted with the electron transport chain in human cells (Blackman et al., 2012). Likewise, a functional screen in yeast identified mitochondrial translation inhibition as the lethality mechanism of the antimicrobial and antileukemic compound tigecycline, and this activity was confirmed in leukemic cells (Skrtić et al., 2011). Finally, Jo et al. (2009a) used yeast to show that a S-adenosylmethionine dependent methyltransferase conferred resistance to various arsenic species, while Ren et al. (2011) showed the corresponding gene in humans (*N6AMT1*) could metabolize arsenic in human urothelial cells. Harari et al. (2013) expanded upon these studies by demonstrating that *N6AMT1* polymorphisms were associated with arsenic methylation in Andean women, and posited that the polymorphisms could be used as susceptibility markers for arsenic toxicity.

## Potential for functional toxicology in other fungi and bacteria

The recent development of the TagModule collection (Oh et al., 2010a), building upon the work of Xu et al. (2007), takes advantage of barcoded transposons to extend the yeast DNA barcoding methodology to a variety of microorganisms. In essence, *in vitro* transposon mutagenesis is utilized to mutagenize a genomic DNA library, and subsequent transformation of barcoded genomic fragments into a compatible unicellular organism allows for genome-wide unbiased screening of chemical-genetic interactions. Akin to the yeast functional process, the barcodes can be amplified from pooled cultures and counted by microarray hybridization or high-throughput sequencing. Oh et al. (2010a) demonstrated the versatility of the TagModule collection by generating tagged mutants in the bacterium *Shewanella oneidensis* MR-1 and the fungal pathogen *Candida albicans*. As a proof of principle, the authors identified *S. oneidensis* mutants with growth deficiencies in minimal media and *C. albicans* mutants sensitive to the antifungal drug clotrimazole (Oh et al., 2010a). The same group reports on additional haploinsufficiency screens in *C. albicans* (Oh et al., 2010b) and *S. oneidensis* (Deutschbauer et al., 2011) encompassing a wide variety of growth conditions and diverse chemical compounds. Furthermore, the method was applied to identify genes important for plant hydrolysate tolerance in *Zymomonas mobilis*, a bacterium with potential for commercial-scale cellulosic ethanol production (Skerker et al., 2013). By facilitating barcoding of mutant or overexpression collections for pooled functional analyses in a range of organisms, the TagModule system can be a valuable tool for toxicity testing.

Alternative approaches utilizing deletion/overexpression strains, or high-throughput sequencing of tagged transposon mutants may have applications relevant to functional toxicology. A signature-tagged mutagenesis strategy permitted parallel analysis of *Cryptococcus neoformans* fungal mutants in experimental infections (Liu et al., 2008), while high-throughput sequencing examined the relative quantities of human gut bacterium *Bacteroides thetaiotaomicron* transposon mutants in wild-type and immunodeficient gnotobiotic mice (Goodman et al., 2009). Relative strain abundance has been quantified in a collection of homozygous *C. albicans* deletion mutants, albeit in a lower-throughput investigation (Noble et al., 2010) than allowed by the TagModule system (Oh et al., 2010a). Overexpression studies can be conducted in *C. albicans*, however the available ORFeome is confined to a few hundred genes (Chauvel et al., 2012). Genome-wide deletion libraries have been constructed in *Escherichia coli* (Baba et al., 2006), *Bacillus subtilis* (Kobayashi et al., 2003), and *Pseudomonas aeruginosa* (Jacobs et al., 2003), with a limited set available in *Salmonella enterica* (Santiviago et al., 2009). Thousands of specific genetic modifications were simultaneously evaluated to quantify population dynamics in various media and growth inhibitors in *E. coli* (Warner et al., 2010). It is conceivable that functional toxicological or even pharmaceutical inquiries can be performed using any of these tools and/or organisms.

## Functional toxicology in eukaryotes of increased complexity

### Overview

Large-scale targeted deletion collections do not currently exist for animal models. Published studies are confined to specific subsets of genes or cellular processes affected by the chemical of interest. Recent advances in knockout technology may make genome-wide functional toxicology possible in complex eukaryotes.

### Studies in cell lines

Until recently, the DT40 B lymphocyte chicken cell lines, because of a hyperactive recombination system, represented the only vertebrate system in which both alleles of a gene could be efficiently disrupted. DT40 cells display a stable phenotype, double in a short period (~8 h), and grow in suspension (Yamazoe et al., 2004; Evans et al., 2010). Thus, DT40 are advantageous in the functional and mechanistic screening of various toxicants. Currently, they are used primarily in the study of genotoxicants, as the majority of the cell lines harbor various individual deletions in DNA repair genes (Ridpath et al., 2007; Yamamoto et al., 2011; Lee et al., 2013). Although other cellular components and processes are not represented in the set of mutants, the cells could become another general resource if additional deletions are generated. However, because the cells are not barcoded, multiplexing or parallel growth screens are not possible.

Genome-wide RNA interference (RNAi) screens have become an important tool in drug discovery (Kiefer et al., 2009), and can be readily applied to functional toxicological studies. RNAi methods, first discovered in *C. elegans* (Fire et al., 1998), exploit existing cellular machinery to destroy the mRNA of a target gene, thus preventing translation and effectively “knocking down” the function of the target gene. RNAi screens in cell lines can be useful to functional toxicology but are experimentally complex, as incomplete knockdown of target genes and off-target effects can complicate execution and analysis (reviewed by North and Vulpe, 2010). The majority of functional genomic applications in human cells rely upon RNAi loss-of-function screens (reviewed by Mullenders and Bernards, 2009). RNAi in *Drosophila* cell lines has been previously utilized to study cellular toxicity—as examples, Eggert et al. (2004) identified small molecules inhibiting the cell cycle, while Zhang et al. (2010) discovered genes that increased the aggregation of mutant Huntingtin proteins. Barcoded short hairpin RNA (shRNA—a method to accomplish RNAi) libraries enable identification of shRNAs that elicit a specific phenotype under toxicant selection. The relative abundance of barcodes in control and treated populations can be measured by hybridization to microarrays (Mullenders and Bernards, 2009) or sequencing (Kimura et al., 2008; Sims et al., 2011). This method is efficient at detecting shRNAs that increase fitness but cannot always discover shRNAs that decrease viability, and furthermore, the process is lengthy and requires significant optimization (Sims et al., 2011). Nevertheless, RNAi-based screens have uncovered human genes whose suppression confers resistance to a wide range of drugs (reviewed by Berns and Bernards, 2012). Finally, a two-stage shRNA screen identified mammalian genetic interactions underlying ricin susceptibility (Bassik et al., 2013).

As noted above, in diploid cells, both chromosomal copies of the gene of interest must be targeted to fully abrogate gene function, an inefficient and laborious step wise process. Thus, an exciting development for the field of functional toxicology is the identification of haploid mouse (Leeb and Wutz, 2011) and near haploid human (Carette et al., 2009) cell lines. Transposon mutagenesis in mouse haploid embryonic stem cells has identified genes required for resistance to 2-amino-6-mercaptopurine (Leeb and Wutz, 2011), the chemotherapeutic 6-thioguanine and the PARP 1/2 inhibitor olaparib (Pettitt et al., 2013), and the bioweapon ricin (Elling et al., 2011). Similarly, the human cells are a derivative of a chronic myeloid leukemia cell line haploid for all chromosomes except chromosome 8. Insertional mutagenesis generated null alleles that have been screened for resistance to host factors used by pathogens (Carette et al., 2009, 2011a; Jae et al., 2013), the cancer drug candidate 3-bromopyruvate (Birsoy et al., 2013), and the ER stressor tunicamycin (Reiling et al., 2011). Especially encouraging is the use of deep sequencing to examine millions of mutant alleles via selection and sequencing of pools of cells (Carette et al., 2011b), an improvement over the laborious analyses of individual clones. These systems promise to advance chemical-genetic studies in human cell lines.

The toolbox for functional toxicology in mammalian cells is expanding. The TALE nuclease architecture has been utilized to regulate mammalian genes and engineer deletions within the endogenous human *NTF3* and *CCR5* genes (Miller et al., 2011). Of particular note is the development of the CRISPR-Cas9 system, which may allow for efficient, large-scale, loss of function screening in mammalian cells (Shalem et al., 2014; Wang et al., 2014). The strategy utilizes a single guide RNA (sgRNA) to direct the Cas9 nuclease to cleave target DNA sequences. With lentiviral delivery of a CRISPR-Cas9 knockout library, sgRNAs serve as distinct barcodes that can be counted via high-throughput sequencing to perform negative and positive selection screening in human cells. One study identified human genes essential for viability in cancer cells, and also discovered human genes necessary for survival in vemurafenib, a RAF inhibitor (Shalem et al., 2014). Others utilized the method in human cell lines to identify genes required for resistance to the nucleotide analog 6-thioguanine and the DNA topoisomerase II inhibitor etoposide (Wang et al., 2014). The simplification and expedition of screening and deletion processes in mammalian cell lines will undoubtedly have considerable ramifications for functional toxicology.

### Studies in whole organisms

The nematode *Caenorhabditis elegans* has been exploited in forward genetic and RNAi screens (reviewed by Leung et al., 2008). Following a round of mutagenesis and selection in a toxicant, next generation sequencing can identify individual *C. elegans* mutants (Doitsidou et al., 2010). Various groups have utilized limited RNAi screens in nematodes to discover genes that, when silenced, confer chemical resistance or susceptibility. Examples include investigations with the herbicide paraquat (Kim and Sun, 2007), whereas gene expression analyses informed RNAi studies with cadmium (Cui et al., 2007) and PCB-52 (Menzel et al., 2007). Although many *C. elegans* homozygous deletion mutants are available through the Caenorhabditis Genetics Center, high-throughput methods to assess survival of all strains at once (in a manner similar to yeast functional profiling) are not available due to the lack of barcode sequences in the mutants. These mutants may prove useful in follow-up analyses of conserved genes and pathways identified by chemical genetic screens in yeasts or bacteria.

Functional toxicology in more complex whole organisms is not straightforward, but new technologies offer encouragement for the future. The zebrafish model is useful in large-scale *in vivo* genetic and chemical studies (Pardo-Martin et al., 2010). Forward chemical genetic screens in zebrafish have identified small molecules inhibiting the cell cycle (Murphey et al., 2006) and examined genes involved in copper-dependent hypopigmentation (Ishizaki et al., 2010). An emerging multidimensional high-throughput *in vivo* approach in embryonic zebrafish, where a wide variety of developmental morphology and neurotoxicity endpoints are rapidly screened to understand chemical toxicity (Truong et al., 2014), indicates that the field of toxicology is advancing in the study of whole organisms. Combining this type of screening strategy with other methodologies such as RNAi, although not currently performed on a large scale basis, could provide additional information related to chemical mechanisms of toxicity.

Traditional approaches for generation of knockout animals in mice remains a time-consuming and costly process that limits its utility in functional toxicology to confirmatory analysis.

However, new systems facilitating deletions in animals can be used to selectively target genes for editing or mutagenesis (Miller et al., 2011; Wilkinson and Wiedenheft, 2014). For example, the site-specific endonuclease TALE-nuclease was used to mediate mutagenesis in mouse zygotes, producing animals with genetic knockouts of the progesterone immunomodulatory binding factor 1 and selenoprotein W, muscle 1 (Sung et al., 2013). The European Conditional Mouse Mutagenesis program (EUCOMM) and the Knockout Mouse Project (KOMP) are two large-scale mouse phenotyping initiatives that aim to provide libraries of knockout animals for further study (Ayadi et al., 2012). The focus of these efforts, however, has been on phenotypic analysis of the mutants rather than the intersection between chemical exposure and genetic background. Knockouts created by these methodologies could expedite analyses of conserved toxicant mechanisms in a variety of animal models. At the present, however, more reasonable is the use of individual animal knockouts or knockdowns to confirm results acquired in less complex systems such as yeast.

## Application of computational analyses to functional toxicology

Inherent in high-throughput functional toxicology methods is the use of computational analyses to decipher toxicant mechanisms of action. Various studies have utilized overenrichment or Cytoscape software (Shannon et al., 2003) to uncover yeast genetic networks affected by a toxicant (Zhou et al., 2009; North et al., 2011; Gaytán et al., 2013a,b; Kaiser et al., 2013). Others, by integrating data from many distinct yeast chemical-genetic datasets, have assembled chemical-phenotype networks that helped identify potential effects elicited by various compounds (Venancio et al., 2010). Without computational analyses, the high-throughput approaches in zebrafish (Truong et al., 2014) or human cell lines (Shalem et al., 2014) would not be possible.

## Challenges with functional toxicology

For a variety of reasons, functional toxicology has its limitations in the range of organisms discussed within this review. Less complex organisms such as yeasts, other fungi, and bacteria possess P450 enzymes (Käppeli, 1986; Urlacher et al., 2004; McLean et al., 2005), but their role in toxicant metabolism is limited. To better understand chemical toxicity to humans, toxicant activation or deactivation may be catalyzed in these experiments by adding S-9 human liver microsomes. Additionally, in unicellular organisms, cell lines, and less complex eukaryotes such as *C. elegans*, one is unable to examine a toxicant's target organs or systemic effects. The discovery of human disease models through orthologous phenotypes may address these concerns. For example, McGary et al. (2010) proposed a yeast model for angiogenesis defects and a *C. elegans* model for breast cancer, based upon analyses of genes shared between the model system and an organism displaying the disease under study. Similar analyses of functional data from unicellular organisms, cell lines, and *C. elegans* may reveal toxicant mechanisms associated with more complex biological processes.

In whole organisms, throughput is the major barrier to progress in functional toxicology. In yeasts and bacteria, barcoded systems allow for the high-throughput examination of thousands of deletion mutants in parallel (Giaever et al., 2002; Oh et al., 2010a). The lack of such methods in whole organisms such as zebrafish or rodent models diminishes throughput, and complicates systematic identification of genes required for resistance to a toxicant. Until high-throughput screens are devised in whole organisms, such systems are invaluable in extending or confirming results gathered in yeasts or bacteria.

## The future of functional toxicology

**Figure 3:**
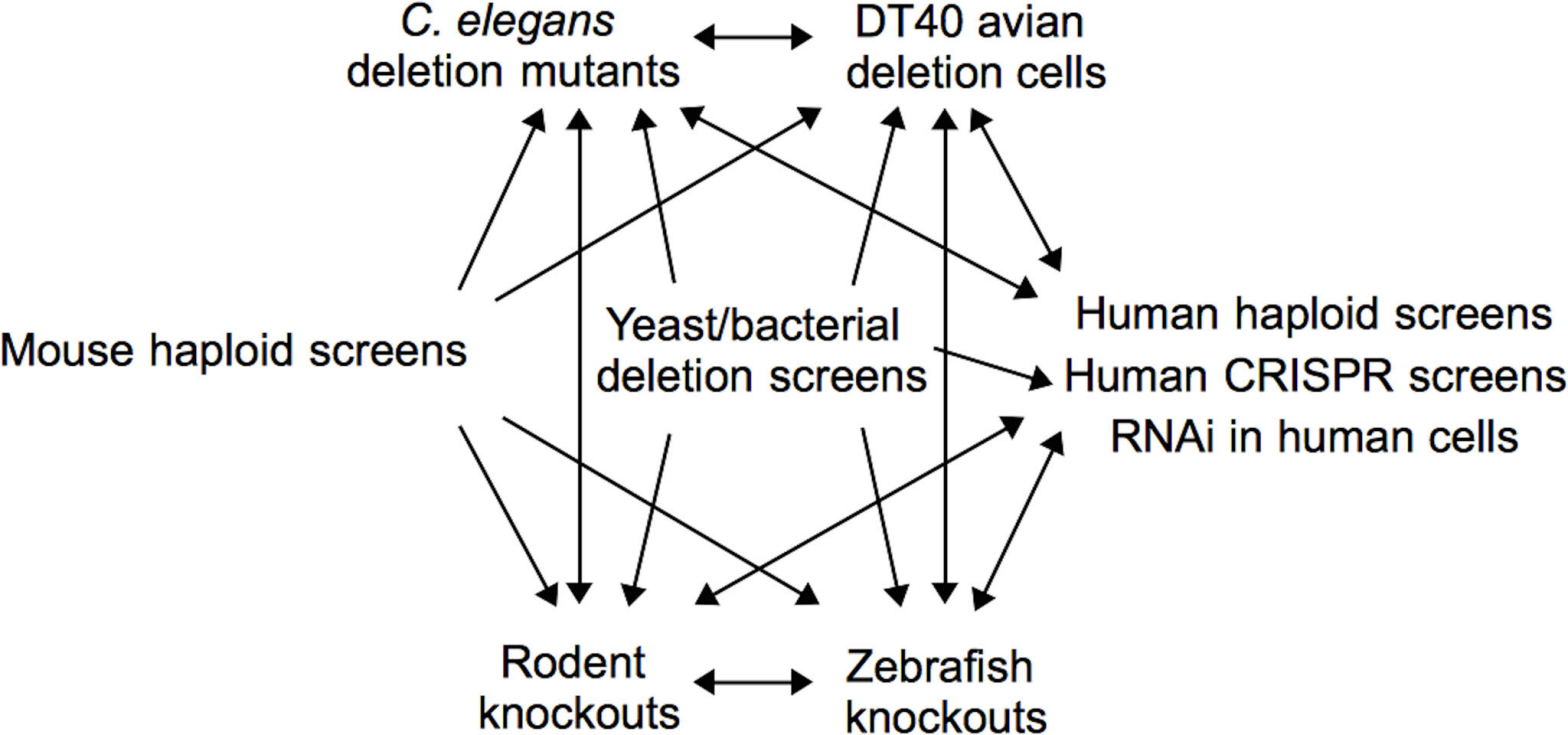
**Integration of functional assays across organisms**. One can use functional tools across a variety of organisms, depending upon the model under study and the end goal of the investigation. For example, one may start with a screen in yeast, mouse, or human cells and extend the analyses to whole organisms such as zebrafish or rodents. Alternatively, one may start with zebrafish mutants or DT40 avian deletion cells and perform follow-up experimentation in human cells or other whole organisms. The many possibilities can advance the future of toxicity testing.

Functional toxicological screening methods, i.e., those that identify genetic requirements for chemical tolerance, are powerful, unbiased tools which provide unique mechanistic insights in the field of toxicology. High-throughput screens of chemicals of concern or unknown toxicity will allow toxicologists to formulate hypotheses related to their corresponding mechanisms and pathways of toxicity. Automation and deep parallel sequencing technologies will unquestionably increase the throughput of functional techniques, but extensive computational resources and knowledge will be required to implement screening systems and analyze the resulting data. Integration of functional assays across a variety of organisms can identify conserved modes of toxicity and direct studies most relevant to human health (Figure 3). The field of functional toxicology is primed to assist toxicologists meet the need for enhanced chemical toxicity testing.

### Conflict of interest statement

The authors declare that the research was conducted in the absence of any commercial or financial relationships that could be construed as a potential conflict of interest.
